# Revealing intra-group immunotherapy response heterogeneity in metastatic urothelial carcinoma through interpretable feature extraction and spectral clustering

**DOI:** 10.3389/fimmu.2025.1629001

**Published:** 2026-01-06

**Authors:** Yoshiyuki Nagumo, Xiucai Ye, Tianyi Shi, Bryan J. Mathis, Tetsuya Sakurai, Hiroyuki Nishiyama

**Affiliations:** 1 Department of Urology, University of Tsukuba, Tsukuba, Japan; 2 Department of Computer Science, University of Tsukuba, Tsukuba, Japan; 3 Department of Cardiovascular Surgery, University of Tsukuba Institute of Medicine, Ibaraki, Japan; 4 Center for Cyber Medicine Research, University of Tsukuba, Ibaraki, Japan

**Keywords:** urothelial carcinoma, immune checkpoint inhibitor, response, biomarker, gene expression, SHAP, spectral clustering

## Abstract

**Introduction:**

Immune checkpoint inhibitors (ICIs) have improved outcomes in metastatic urothelial carcinoma (mUC) but clinical responses remain highly heterogenous. Traditional binary classification of response overlooks clinically relevant variability within each group but a more detailed understanding of intra-group heterogeneity may support subclass-specific therapeutic strategies.

**Methods:**

We developed a novel analysis framework that integrates interpretable feature extraction and spectral clustering to identify patient subclasses associated with heterogeneous responses to ICIs. This method was applied to tumor transcriptomic data from the IMvigor210 cohort (n = 298), comprising mUC patients treated with atezolizumab. Interpretable features based on SHapley Additive exPlanations (SHAP) were computed from a response classification model to quantify patient-level gene contributions, which were then used for spectral clustering. An independent cohort (GSE176307, n = 88) was used for external validation.

**Results:**

This approach identified four patient clusters with distinct immune phenotypes and response patterns. Cluster 3 (92.3% responders) showed an inflamed phenotype with high PD-L1 expression, T cell activation, and TP53 mutations. Cluster 1 (100% non-responders) displayed an immune-desert phenotype with FGFR3 mutations and elevated TGF-β signaling. Cluster 2 was more heterogeneous, containing two subgroups (Sub 1 and Sub 2) with differing immune activity and immunosuppressive gene expression, corresponding to response rates of 23.2% and 77.3%, respectively. Similar patterns were observed in the validation cohort.

**Conclusions:**

Our framework, which combines SHAP-based interpretable feature extraction with spectral clustering, revealed subclass-level heterogeneity in ICI response, highlighting biologically distinct immune subclasses. This approach may facilitate the development of subclass-specific therapeutic strategies.

## 1 Introduction

Immune checkpoint inhibitors (ICIs) targeting the PD-L1/PD-1 pathway, such as avelumab and pembrolizumab, have significantly improved survival outcomes in patients with metastatic urothelial carcinoma (mUC) compared to conventional chemotherapy (1, 2). Furthermore, enfortumab vedotin (targeting Nectin-4) combined with pembrolizumab, as well as cisplatin-based chemotherapy combined with nivolumab (another anti-PD-1 inhibitor), have demonstrated markedly improved outcomes as first-line treatments, highlighting the growing importance of immunotherapy in the management of mUC (3, 4). Although ICI-based combination therapies, such as those involving enfortumab vedotin or chemotherapy, have shown high objective response rates of 57-67% (3, 4), ICI monotherapy yields a response rate of only 10-20% (1, 2). This discrepancy suggests that true ICI responders are limited to a small subset of patients, highlighting the need to better understand the underlying response mechanisms and develop strategies for identifying cases of greatest benefit.

Previously identified tissue biomarkers, such as PD-L1 expression, tumor mutation burden (TMB), and gene expression-based molecular subtypes, have been used to classify responders to ICIs (5–7). Although each is contributive to treatment response, these biomarkers are poorly compatible with daily clinical practice and have limited overall predictive accuracy. In addition, conventional analytical approaches from which such biomarkers are derived may not fully capture the complex, heterogenous nature of T cell interactions that drive the tumor microenvironment and mechanisms of progressive therapy resistance. These issues highlight the need for novel analytical frameworks that extend beyond the identification of binary response/non-response into biologically meaningful patient subclasses to provide individualized snapshots of predicted treatment response.

Several studies have employed clustering techniques to stratify ICI responders based on the tumor immune milieu and localized gene expression (8, 9). However, current reports often rely on unsupervised methods that may not directly incorporate treatment outcomes while the integration of interpretable machine learning techniques, such as SHapley Additive exPlanations (SHAP), may better extract biologically relevant, patient-specific features to enhance stratification (10, 11). Notably, recent studies have applied SHAP to identify key biological factors associated with cancer presence or treatment response in some settings, including the gut-microbiome and microRNA profiles (12–15). These findings highlight the potential for SHAP to delineate heterogeneity in treatment response patterns.

Based on these advances, we here present an extraction of interpretable features from gene expression data by computing patient-level SHAP values. To investigate this, we developed a classification model using ICI response as the outcome label and applied SHAP to quantify gene-level contributions to individual predictions. We then performed principal component analysis (PCA)-based dimensionality reduction and spectral clustering using SHAP values to identify clinically and immunologically distinct patient subclasses, including subgroups within conventionally defined responder or non-responder groups. Through this approach, our study aims to propose a new framework for understanding response heterogeneity and guiding subclass-specific immunotherapeutic strategies in mUC.

## 2 Materials and methods

### 2.1 Data acquisition

We obtained transcriptomic, gene mutation, and clinical information data from mUC patients (n = 298) treated with atezolizumab from the publicly available IMvigor210CoreBiologies dataset (http://research-pub.gene.com/IMvigor210CoreBiologies/) (16). Patients were categorized into responders (complete or partial response, n = 131) or non-responders (stable or progressive disease, n = 167) according to Response Evaluation Criteria in Solid Tumor (RECIST) ver1.1 (17). An external cohort, GSE176307 (n = 88), which includes transcriptomic and clinical data from mUC patients treated with ICIs, was used for validation purposes (18). In this cohort, patients received anti-PD-1 or anti-PD-L1 inhibitors, including pembrolizumab or atezolizumab. Most patients had previously received platinum-based chemotherapy before ICI administration, whereas a small subset received ICIs as first-line treatment. Of the 88 patients, 68 were classified as responders and 20 as non-responders.

### 2.2 Data processing

For the IMvigor210 cohort, raw RNA sequencing counts were first normalized to transcripts per million (TPM) using gene length–adjusted counts. The TPM values were then log_2_-transformed to prepare the data for downstream analysis. To select highly variable genes between responders and non-responders, we next performed differentially expressed gene (DEG) analysis using the limma package (19) in R (version 4.4.1; R Foundation, Vienna, Austria). Genes with a false discovery rate (FDR) < 0.05 and fold change > 1.3 were considered DEGs (n = 182). This moderately permissive threshold was selected to retain a sufficient input dimensionality for subsequent downstream analyses. For the GSE176307 cohort, TPM-normalized gene expression data were similarly log_2_-transformed.

### 2.3 Interpretable feature extraction

To extract interpretable features in the IMvigor210 cohort, we constructed a supervised classification model to distinguish responders from non-responders using the 182 DEGs as input features. The model was trained using the XGBoost package in Python (20), with parameters tuned to prevent overfitting and enhance model performance reliability. SHAP values were computed for each patient to extract interpretable features, generating a matrix of gene-level importance scores that represented patient-specific molecular profiles.

### 2.4 Cluster analysis

After extracting interpretable features, we performed PCA-based dimensionality reduction on the SHAP values. The number of principal components was determined based on the standard deviation of the components, retaining those that contributed substantially to the overall variance. Spectral clustering was then applied to identify subclusters. To construct the similarity matrix for spectral clustering, we utilized a shared nearest neighbor (SNN) approach (21), in which the similarity between two samples is defined by the number of neighbors they share. The number of nearest neighbors was set dynamically: a minimum of 5 was enforced to ensure robustness while the maximum was scaled to the size of the dataset.

### 2.5 Enrichment analysis

Gene Ontology (GO) enrichment analysis for biological processes (BP) was performed using the enrichGO function from the clusterProfiler package in R (22). Input genes were based on symbols, and the org.Hs.eg.db annotation database was used. GO terms with adjusted p-values (Benjamini–Hochberg method) and q-values < 0.05 were considered significantly enriched.

### 2.6 Statistical analysis

For survival analysis, overall survival (OS) was evaluated using the Kaplan–Meier method, and differences between patient subclasses were assessed with the log-rank test. Multivariate Cox proportional hazards models were applied to evaluate the prognostic impact of patient subclass after adjusting for clinical covariates. Continuous variables were divided into high and low groups based on the median value. All statistical analyses were performed using R (version 4.4.1), with p < 0.05 considered statistically significant.

### 2.7 Data and code availability

The code used for this manuscript is available at the following URL: https://github.com/Tianyi-Shi-Tsukuba/Metastatic-Urothelial-Carcinoma-Clustering-SHAP. In addition, we have developed a graphical user interface (GUI)-based tool that implements the SHAP value–based clustering algorithm described in this study. This tool is publicly available at the following URL: https://metastatic-urothelial-carcinoma-clustering-shap-msyyxk86ut8cp7.streamlit.app/.

## 3 Result

### 3.1 Conventional unsupervised clustering

**Figure 1:**
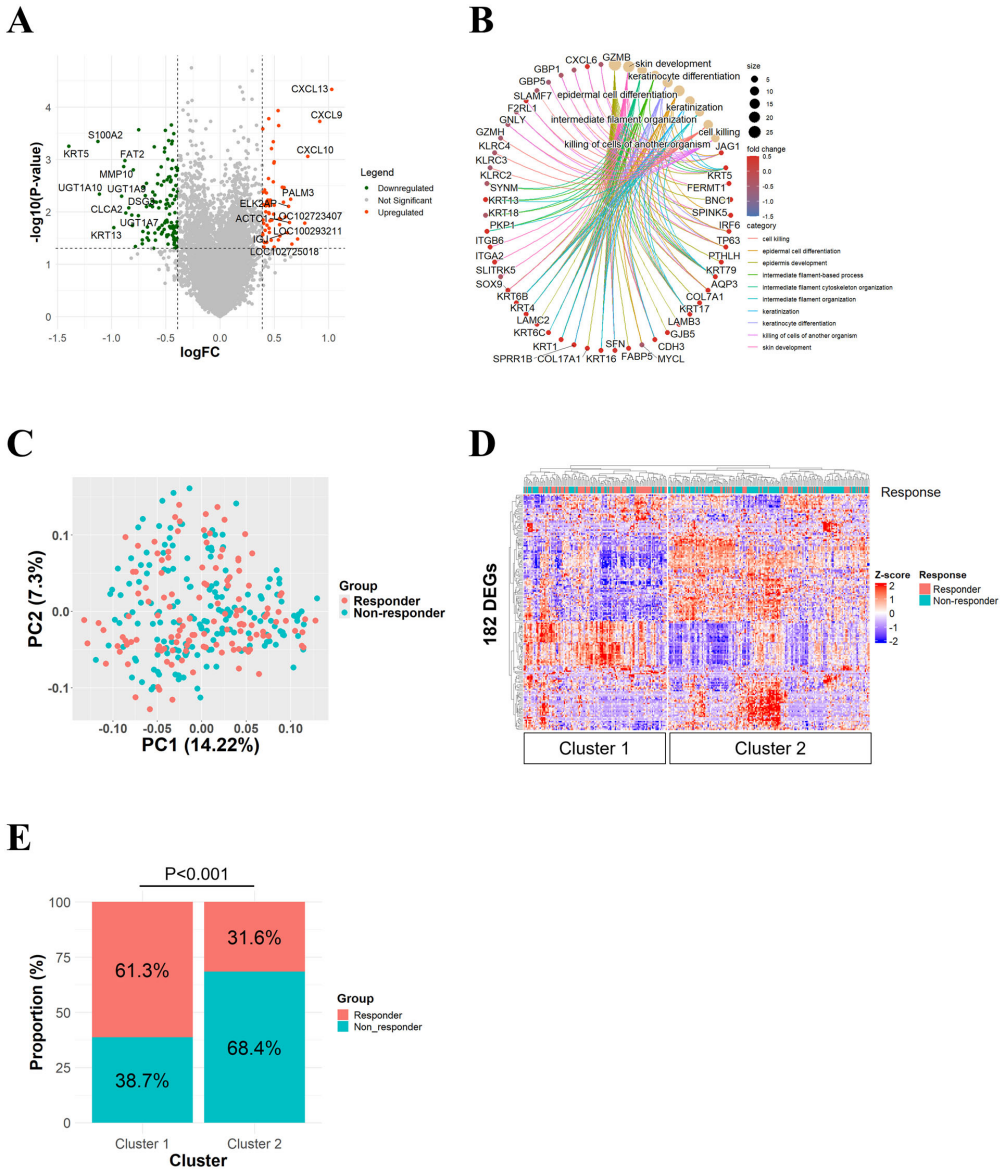
Conventional clustering methods using 182 DEGs between responders and non-responders in the IMVigor210 cohort were unable to clearly identify patient subclasses associated with immunotherapy response. **(A)** A volcano plot showing 182 DEGs between responders and non-responders. **(B)** A network plot showing representative enriched pathways. **(C)** A PCA plot showing the distribution of responder (pink) and non-responder (blue) samples. **(D)** A hierarchical clustering heatmap showing the division of samples into two clusters. **(E)** A bar plot showing proportional distribution of responders and non-responders across the two clusters.

Initially, we performed DEG analysis to investigate the differences in gene expression profiles between responders (n=131) and non-responders (n=167) in the IMvigor210 dataset (n=298). A total of 182 DEGs were extracted, of which 121 were upregulated and 61 were downregulated in responders (**Figure 1A**). Notably, upregulated genes included those involved in T-cell immune response pathways, such as CXCL13, CXCL10, and CXCL9. The biological functions of these DEGs were predominantly associated with enhanced T-cell-mediated cytotoxicity (**Figure 1B**).

Next, we evaluated whether these 182 DEGs could classify responders using conventional clustering methods. As a conventional clustering approach, hierarchical clustering was performed using Pearson distance and complete linkage with ComplexHeatmap package in R (23). PCA failed to demonstrate clear separation trends between responders and non-responders (**Figure 1C**). In contrast, hierarchical clustering revealed two major clusters, with Cluster 1 having a significantly higher proportion of responders compared to Cluster 2 (61.3% vs. 31.6%, p < 0.001) (**Figures 1D, E**). Similar trends were observed by k-means clustering **(Supplementary Figures 1A, B)**.

These results suggest that while conventional clustering methods do classify patient subclasses with relatively higher response rates, responder/non-responder co-presence within each cluster due to the heterogeneity of immunotherapy responses remains a key limitation of this approach.

### 3.2 PCA plot of SHAP values in our framework

Since conventional clustering methods using DEGs are inadequate for subclassification, we hypothesized the presence of gene expression characteristics specific to responders. We thus introduced a novel analysis framework that extracts interpretable features using SHAP derived from a classification model trained on response labels. This approach aims to reveal gene expression profiles and potential subclass characteristics associated with response.

**Figure 2:**
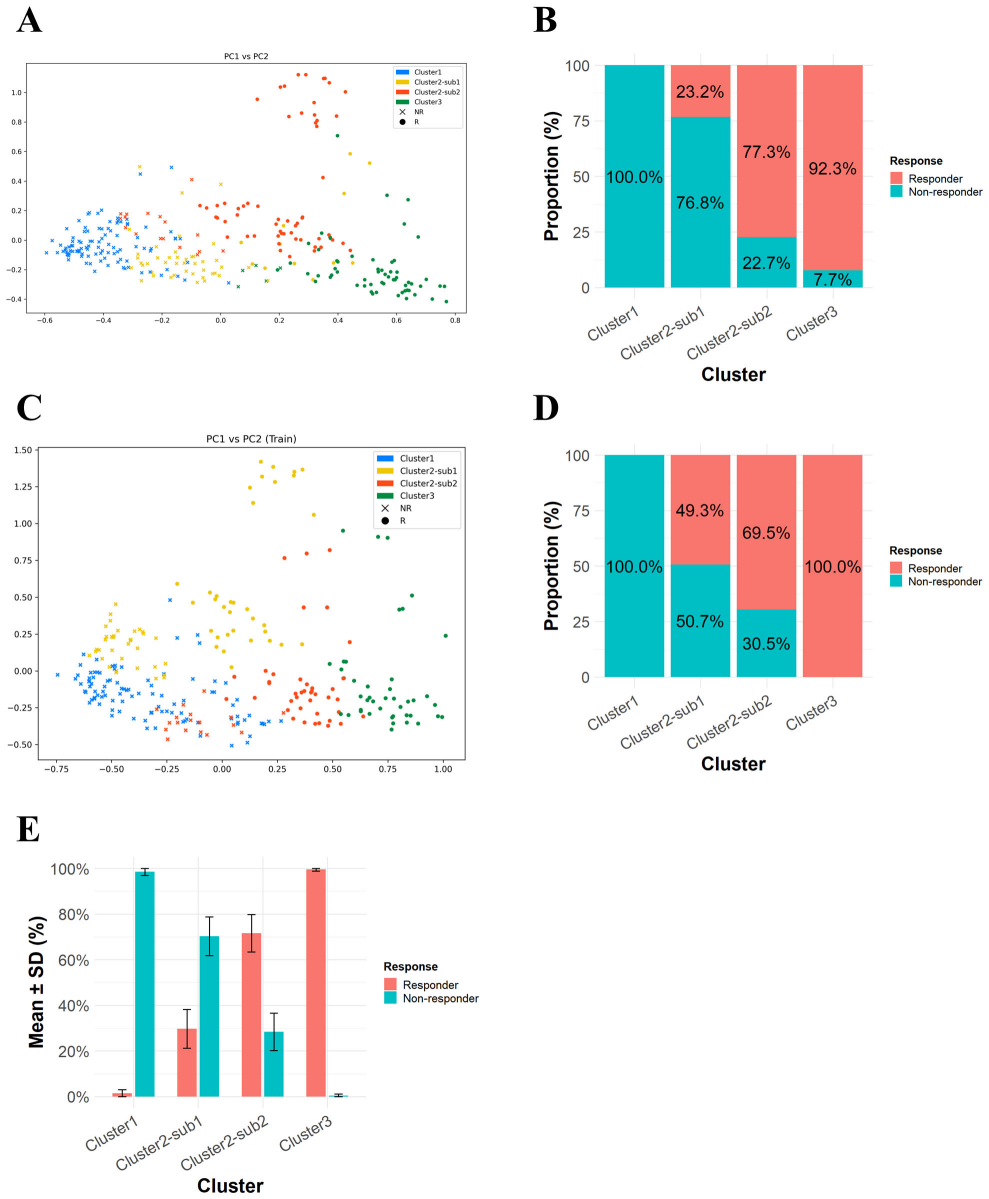
PCA plot of SHAP values for 182 DEGs highlights potential patient subclasses with distinct response profiles. **(A)** A PCA plot of SHAP values for 298 patients. **(B)** A bar plot showing response distributions across the four clusters. **(C)** A PCA plot of SHAP values for training data. **(D)** A bar plot showing response distributions across patient clusters in a random 90% subsamples. **(E)** A bar plot showing mean response distributions across patient clusters in ten random 90% subsamples.

As shown in **Figure 2A**, a PCA plot of SHAP values revealed three major clusters. Notably, Cluster 2 could be further divided into two subclusters: Sub 1, which was closer to Cluster 1, and Sub 2, which was closer to Cluster 3. Regarding response profiles, the proportion of progressive disease (PD) cases was high in Cluster 1 and Cluster 2-Sub 1, at 100% and 76.8%, respectively (**Figure 2B**). In contrast, the proportions of PD were much lower in Cluster 2-Sub 2 and Cluster 3, at 22.7% and 7.7%, respectively.

To evaluate the robustness of the proposed clustering framework, we carried out a stability analysis using random subsampling. In this procedure, 90% of the samples were randomly selected from the full IMvigor210 dataset ten times, and clustering was independently carried out for each subsample using the same algorithm. For each iteration, we checked whether the balance between responders and non-responders within the obtained clusters remained consistent. As shown in **Figure 2C**, a PCA plot from one random subsample displayed a distribution pattern comparable to that derived from the complete dataset (**Figure 2A**). The corresponding bar plot (**Figure 2D**) presents the response proportions across clusters in that subsample. When the results from all ten repetitions were combined, the averaged response distributions (**Figure 2E**) varied only slightly among clusters, suggesting that the grouping and associated response profiles were not sensitive to sampling fluctuations. These results suggest that our SHAP-based clustering method can reproducibly identify patient subclasses, reflecting intra-group heterogeneity in ICI response and extending beyond conventional binary response categories (responder or non-responder).

### 3.3 Potential patient subclasses exhibited distinct characteristics

**Figure 3:**
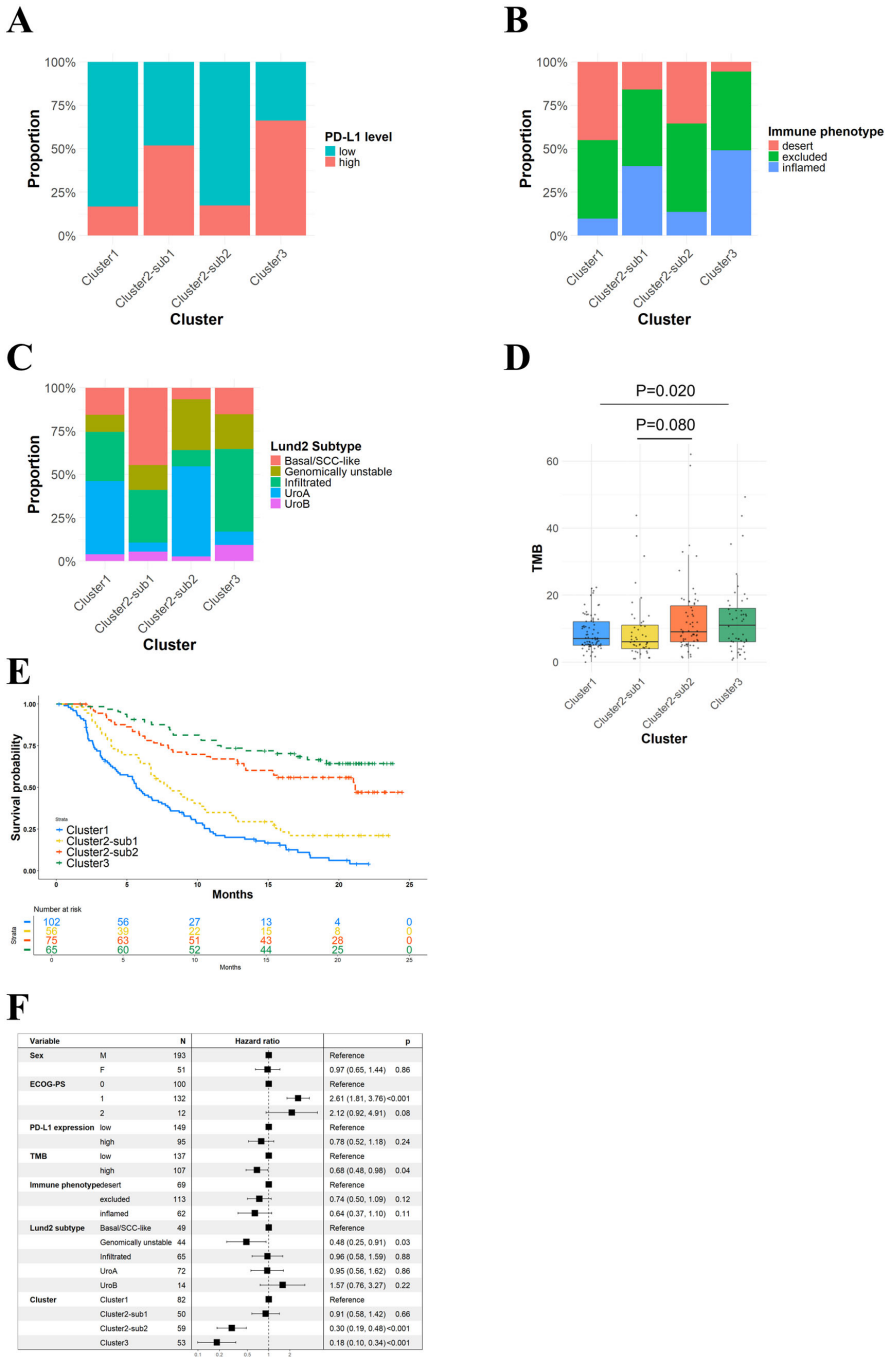
Potential patient subclasses exhibited distinct characteristics with clinical significance including differences in patient prognoses. **(A)** A bar plot showing proportional distribution of PD-L1 expression levels across patient subclasses. **(B)** A bar plot showing proportional distribution of immune phenotypes. **(C)** A bar plot showing proportional distribution of Lund 2 subtypes. **(D)** A box plot showing tumor mutation burden across patient subclasses. **(E)** Kaplan-Meier curves of overall survival for patient subclasses. **(F)** A forest plot of hazard ratios for overall survival associated with clinical and molecular factors.

We next examined the clinical and tumor characteristics of the potential patient subclasses identified by our analysis framework. Regarding PD-L1 expression levels, the proportion of high PD-L1 expression was significantly higher in Cluster 3 than Cluster 1 (66.2% vs. 17.0%, p < 0.001) (**Figure 3A**). Similarly, within the subclasses of Cluster 2, the proportion of high PD-L1 expression was significantly higher in Cluster 2-Sub 1 compared to Cluster 2-Sub 2 (51.8% vs. 17.3%, p < 0.001). For immune phenotype, Cluster 1 exhibited a significantly higher proportion of desert phenotype compared to Cluster 3 (45.1% vs. 5.7%, p < 0.001), while the inflamed phenotype was significantly less frequent in Cluster 1 (9.7% vs. 49.1%, p < 0.001) (**Figure 3B**). Within Cluster 2, Cluster 2-Sub 1 showed a significantly lower proportion of desert phenotype (16.0% vs. 35.6%, p = 0.029) and a significantly higher proportion of inflamed phenotype (40.0% vs. 13.6%, p = 0.002) compared to Cluster 2-Sub 2.

For molecular subtypes (LundTax extended classification), Cluster 1 had a significantly higher proportion of Uro A subtype compared to Cluster 3 (42.2% vs. 7.7%, p < 0.001) and a significantly lower proportion of the Infiltrated subtype (28.4% vs. 47.7%, p = 0.013) (**Figure 3C**). Additionally, Cluster 2-Sub 1 had the highest proportion of Basal/SCC-like subtype (44.6%), while Cluster 2-Sub 2 had the highest proportion of Genomically Unstable (GU) subtype (29.3%). Regarding mean tumor mutational burden (TMB) levels, Cluster 1 exhibited significantly lower TMB compared to Cluster 3 (8.7 vs. 12.7, p = 0.020). No significant differences were observed between the subclasses of Cluster 2 (9.3 vs. 12.8, p = 0.080) (**Figure 3D**).

Furthermore, patient prognoses were analyzed within each subclass. As shown in **Figure 3E**, the median OS rates for Cluster 3, Cluster 2-Sub 2, Cluster 2-Sub 1, and Cluster 1 was: Not Reached, 21.1 months (95% CI: 13.4-NA), 7.9 months (95% CI: 6.7-12.4), and 5.6 months (95% CI: 4.4-7.9), respectively. In multivariate analyses of OS, ECOG-PS was identified as an independent poor prognostic factor while, in contrast, higher TMB levels, the Genomically Unstable subtype, Cluster 2-Sub 2, and Cluster 3 were independent favorable prognostic factors (**Figure 3E**).

These results suggest that the patient subclasses identified by our SHAP-based clustering method exhibit distinct combinations of clinical, molecular, and prognostic outcomes, highlighting their clinical utility for predictive reproducibility.

### 3.4 A further DEG analysis between the patient subclasses identified potential genes associated with immunotherapy response

**Figure 4:**
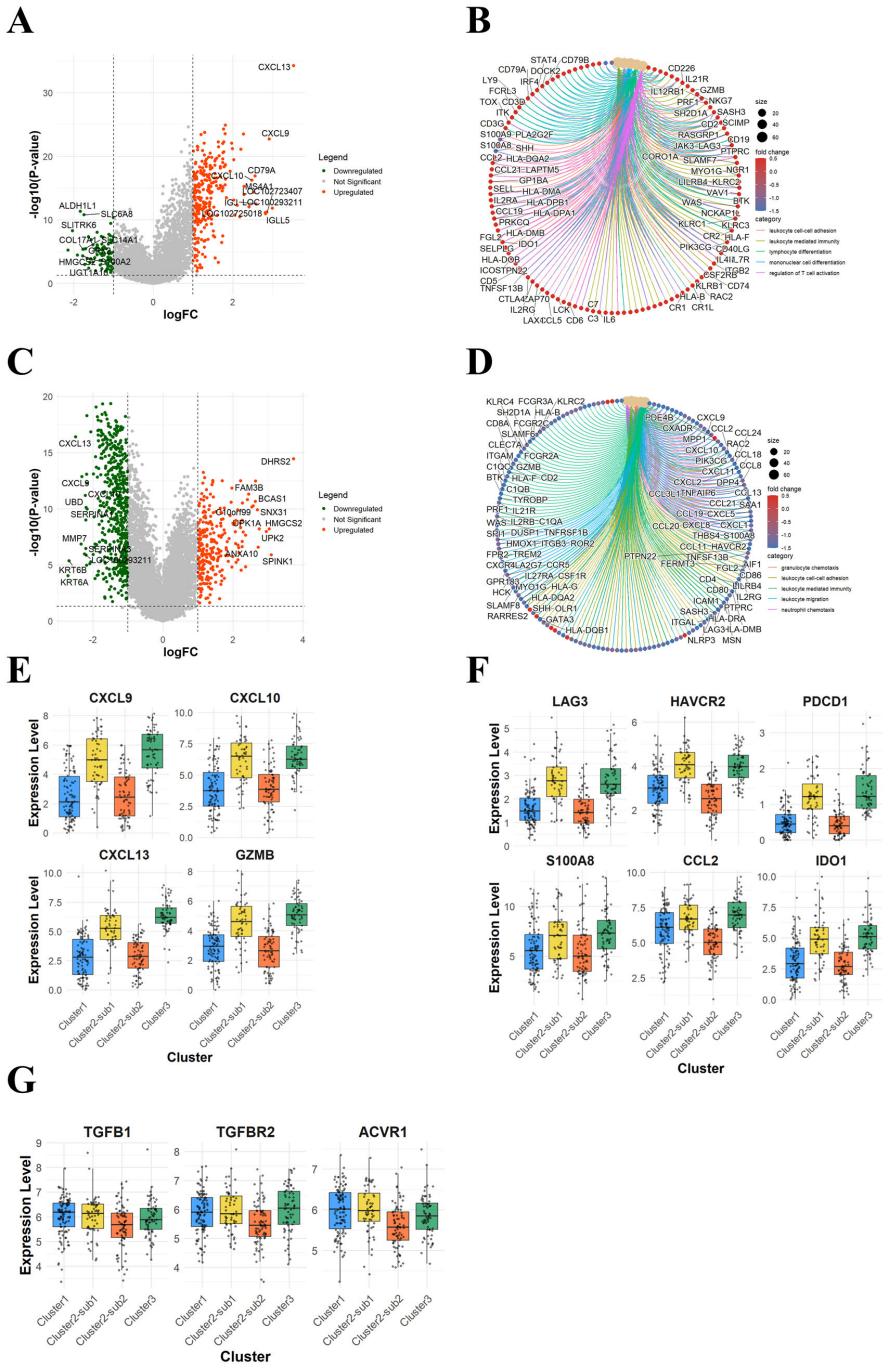
A further DEG analysis between the patient subclasses identified potential genes associated with immunotherapy response. **(A)** A volcano plot showing 365 DEGs between Cluster 1 and Cluster 3. **(B)** A heatmap of 365 DEGs stratified by clusters. **(C)** A volcano plot showing 808 DEGs between Cluster 2-Sub 1 and Cluster 2-Sub 2. **(D)** A network plot of enriched pathways and associated genes for 808 DEGs between Cluster 2-Sub 1 and Cluster 2-Sub 2. **(E)** Box plots showing T-cell activation-related gene expression levels across the four clusters. **(F)** Box plots showing immunosuppressive gene expression levels across the four clusters. **(G)** Box plots showing TGF-β-related gene expression levels across the four clusters.

To identify discrete genes associated with therapeutic response between responders and non-responders, we conducted further DEG analyses between Clusters 1 and 3. A total of 365 DEGs were extracted, with 277 genes upregulated and 88 genes downregulated in Cluster 3 (**Figure 4A**). Functional analysis of these DEGs revealed significant enrichment in pathways related to T-cell activation (**Figure 4B**). Within Cluster 2, the clinical/tumor characteristics, response distributions, and prognoses differed significantly between Subclusters 1 and 2, suggesting distinct transcriptomic profiles may underlie their treatment response phenotypes. A total of 808 DEGs were identified between Cluster 2-Sub 1 and Cluster 2-Sub 2, with 271 genes upregulated and 537 genes downregulated in Cluster 2-Sub 2 (**Figure 4C**). Functional enrichment analysis showed that genes downregulated in Cluster 2-Sub 2, compared to Cluster 2-Sub 1, were predominantly associated with inflammatory immune responses involving neutrophils and other myeloid cells that contribute to immunosuppression within the tumor microenvironment (**Figure 4D**).

Next, we examined the expression levels of T-cell-related genes that are characteristic of response, as identified in the comparison between Clusters 1 and 3, across all four clusters. Interestingly, genes such as CXCL9, CXCL10, CXCL13, and GZMB were also highly expressed in Cluster 2-Sub 1, a non-responder subclass (**Figure 4E**). Similarly, genes associated with immune-suppressive myeloid inflammation and T-cell exhaustion, identified in the comparison between Cluster 2-Sub 1 and Cluster 2-Sub 2, were also expressed at high levels in Cluster 3, a responder subclass (**Figure 4F**). Given the previous study in the IMvigor210 cohort suggesting the involvement of TGF-β signaling in non-response (16), we analyzed the expression levels of TGF-β-related genes across the four clusters. Consistent with prior findings, these genes were more highly expressed in non-responder subclasses (Cluster 1 and Cluster 2-Sub 1) compared to responder subclasses (Cluster 3 and Cluster 2-Sub 2) (**Figure 4G**).

These findings suggest that further DEG analysis of SHAP cluster-derived patient subclasses reveals distinct patterns of immune activation and suppression. Specifically, two responder subclasses were identified: Cluster 2-Sub 2, characterized by low expression of both immune activation and suppressive signals, and Cluster 3, marked by high expression of both signals. Similarly, two non-responder subclasses were observed: Cluster 1, exhibiting minimal immune activation and high TGF-*β* signaling, and Cluster 2-Sub 1, showing robust immune activation alongside immunosuppressive signals. Together, these results highlight the heterogeneous nature of the immunotherapy response milieu within the tumor microenvironment.

### 3.5 Distinct transcriptomic, genomic, and clinical profiles define each patient subclass

**Figure 5:**
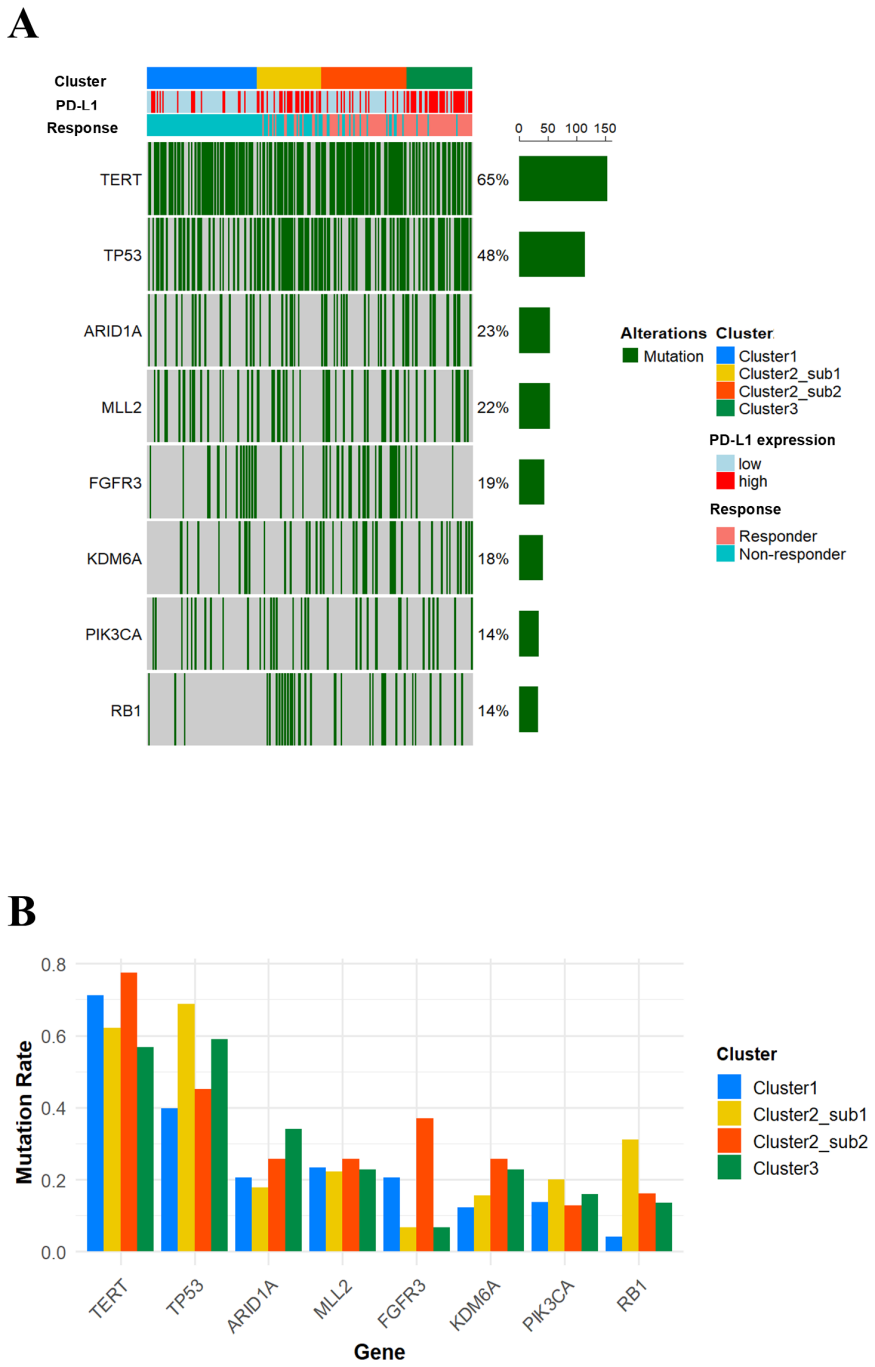
Mutation profiles stratified by each cluster. **(A)** An oncoprint illustrating gene mutations with a frequency of 10% or higher. **(B)** A bar plot showing the differences in the frequency of the indicated gene mutations across the four clusters.

Next, we compared genomic mutation profiles across the four clusters. As shown in **Figures 5A, 5** and 5B, TP53 mutations were more frequently observed in Cluster 2-Sub 1 and Cluster 3, while FGFR3 mutations were more common in Cluster 1 and Cluster 2-Sub 2. Additionally, RB1 mutations were particularly frequent in Cluster 2-Sub 1. These findings suggest that genomic mutation profiles, similar to transcriptomic profiles, are molecularly distinct across each subclass.

As summarized in **Table 1**, the tumor characteristics of the identified subclasses highlight distinct genomic and immune profiles that correlate with immunotherapy outcomes. Cluster 1 (characterized by FGFR3 mutations, low PD-L1 expression, and an immune-desert phenotype) demonstrated consistently poor outcomes, with minimal T-cell activation and high TGFβ expression, potentially driving immune exclusion. Cluster 2-Sub 1, despite being a “hot” (inflamed) tumor with high PD-L1 expression and robust T-cell activation, was also associated with poor prognoses. This is likely driven by the high frequency of RB1 mutations, elevated TGF-β expression, and increased immunosuppressive gene expression, all of which contribute to a profoundly suppressive tumor microenvironment that ablates T cell anti-tumor activity. In contrast, Cluster 2-Sub 2 combined FGFR3 mutations, high TMB, and limited immune suppression, highlighting the importance of a less suppressive tumor microenvironment. This unique combination correlates with significantly improved response rates and prolonged survival, suggesting that the weakened immunosuppression may enhance the immune checkpoint blockade. Lastly, Cluster 3, defined by frequent TP53 mutations, high TMB, and an inflamed immune phenotype, achieved the best therapeutic outcomes, consistent with a hyper-inflamed tumor state that is highly responsive to immune checkpoint blockade. Collectively, these findings highlight the intra-group heterogeneity of immunotherapy responses in mUC, revealing how diverse combinations of genomic alterations and immune-related features reliably associate with treatment outcomes, even among patients classified as responders.

**Table 1:** Characteristics of each cluster.

Characteristics	Cluster1	Cluster2-Sub 1	Cluster2-Sub 2	Cluster3
No. of patients (%)	102 (34.2)	56 (18.8)	75 (25.2)	65 (21.8)
Response rate (%)	0.0	23.2	77.3	92.3
Median overall survival (month, range)	5.7 (4.4-7.9)	7.9 (6.7-12.4)	21.2 (13.4-NA)	NA (NA-NA)
PD-L1 expression level	Low	High	Low	High
Immune phenotype	Desert	Excluded/inflamed	Desert	Excluded/inflamed
LundTax subtype	Uro A	Basal/SCC-like	Genomically unstable	Infiltrated
TMB level	Low	Low	High	High
T cell activation gene expression level	Low	High	Low	High
Immunosuppressive gene expression level	Low	High	Low	High
TGFB1 expression level	High	High	Low	Low
TP53 mutation frequency	Low	High	Low	High
FGFR3 mutation frequency	High	Low	High	Low
RB1 mutation frequency	Low	High	Low	Low
Features	Immune-desert tumors	Immune-excluded tumors with high immune suppression	Immune-desert tumor with high TMB and low immune suppression	Immune-infiltrated tumors with high TMB and T-cell activation alongside immune suppression

### 3.6 Exploratory classification based on selected immunogenomic parameters

**Figure 6:**
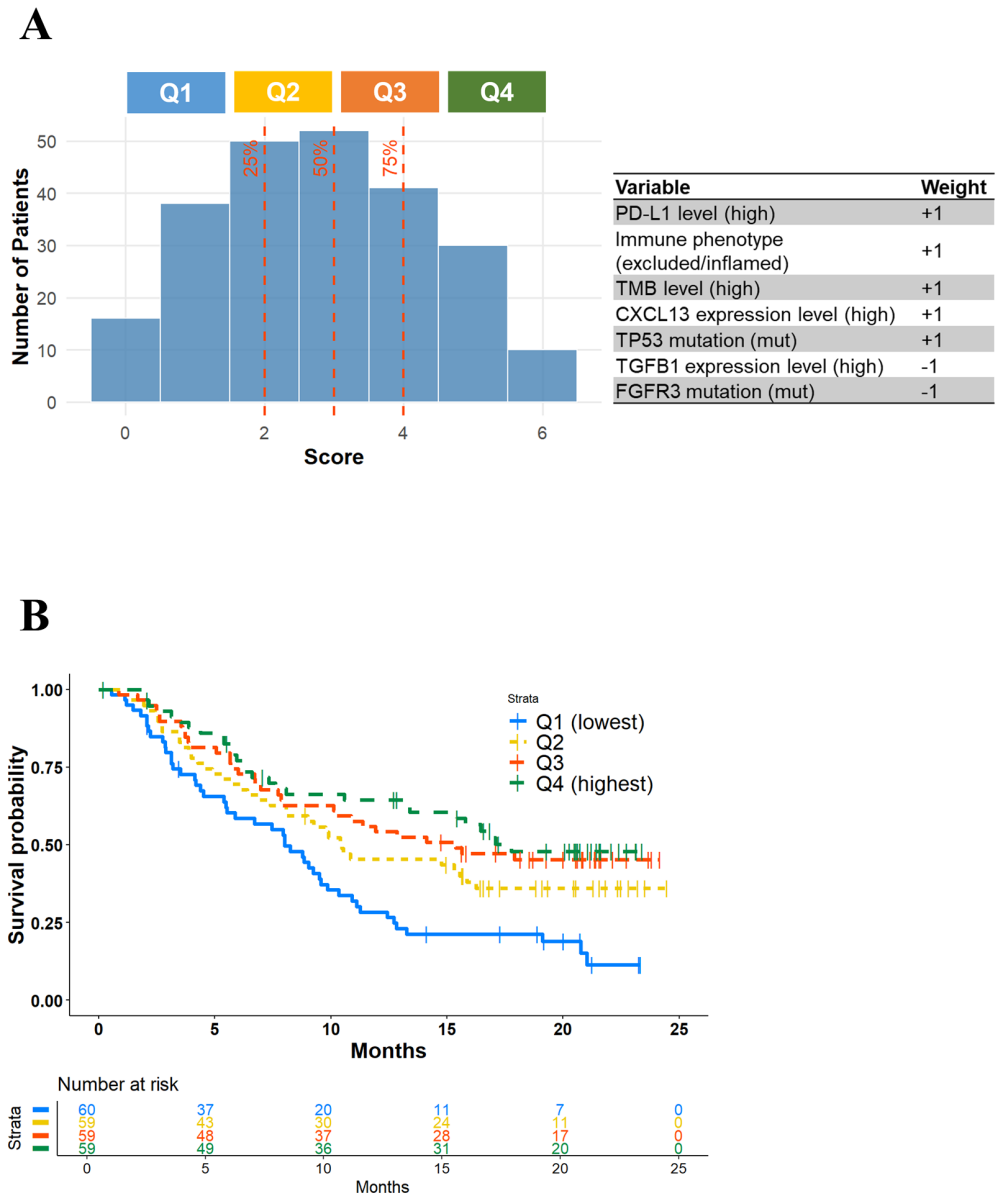
Patient classification based on selected immunogenic parameters. **(A)** A histogram of composite scores for each patient and selected immunogenic parameters. **(B)** Kaplan-Meier curves of overall survival for patient subclasses.

Next, to explore the feasibility of clinically applicable patient subclassification, we examined a simplified approach using selected immunogenomic parameters identified by our framework. Based on the characteristic features of each cluster (**Table 1**), we selected PD-L1 level (high), immune phenotype (excluded/inflamed), TMB level (high), CXCL13 expression level (high), and TP53 mutation (mut) as immune activation factors, while TGFB1 expression level (high) and FGFR3 mutation (mut) were defined as immune suppression factors. Using these seven selected parameters, we calculated a composite score for each patient and classified them into four groups according to quartiles (**Figure 6A**). As shown in **Figure 6B**, OS in the Q4 group, which had the highest scores, was longer compared to the Q1 group with the lowest scores, showing a pattern similar to that observed between Cluster 3 and Cluster 1 in **Figure 3E**. Interestingly, the OS difference observed between the Q2 and the Q3 groups also resembled that between Cluster 2-Sub1 and Cluster 2-Sub2, suggesting that the simplified scoring system may partly capture the intra-group heterogeneity of immunotherapy responses. However, its discriminative resolution appears limited and further investigation, including optimization of parameter selection and weighting, will be necessary to improve its clinical applicability.

### 3.7 Validation of SHAP-based clustering using potential immunotherapy-associated genes in an independent cohort

Finally, we applied our analysis framework to an independent cohort to validate the applicability and consistency of our novel clustering approach. To evaluate whether the response-associated subclasses identified in the IMvigor210 cohort could be reproduced in an independent cohort, we utilized the 365 DEGs between Cluster 1 (100% non-responders) vs. Cluster 3 (92.3% responders) in the IMvigor210 dataset. In the GSE176307 dataset (n=88), 359 of these 365 DEGs overlapped.

**Figure 7:**
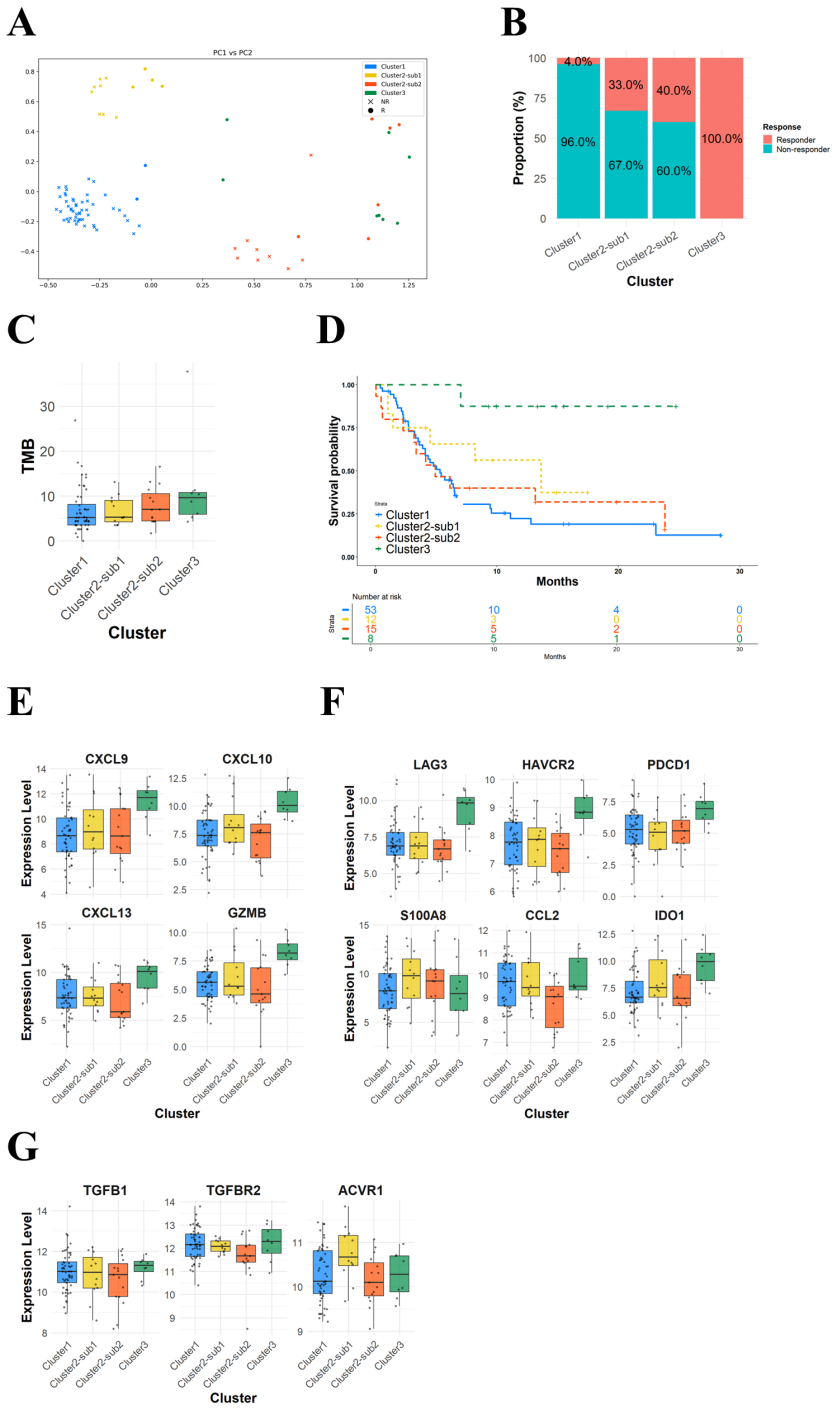
PCA plot of SHAP values for immunotherapy response associated genes highlights potential patient subclasses with distinct response profiles in an independent cohort. **(A)** A PCA plot of SHAP values for 88 patients. **(B)** A bar plot showing response distributions across the four clusters. **(C)** A box plot showing tumor mutation burden across patient subclasses. **(D)** Kaplan-Meier curves of overall survival for patient subclasses. **(E)** Box plots showing T-cell activation-related gene expression levels across the four clusters. **(F)** Box plots showing immunosuppressive gene expression levels across the four clusters. **(G)** Box plots showing TGF-β-related gene expression levels across the four clusters.

Next, we calculated SHAP values for these 359 genes and performed PCA-based dimensionality reduction and spectral clustering. As shown in **Figure 7A**, compared to the IMvigor210 dataset, the PCA plot of SHAP values revealed four distinct clusters consistent with the patterns and response distributions observed in the IMvigor210 dataset (**Figure 7B**). Regarding the TMB levels, although Cluster 3 showed a higher mean TMB than Cluster 1 (11.8 vs. 6.8), the difference was not statistically significant (p=0.237) (**Figure 7C**) but, in terms of OS, the median OS was longer in Cluster 3 compared to Cluster 1 (Not Reached vs. 5.3 months), even if statistical significance was not assessed due to the inclusion of low patient number clusters (**Figure 7D**). Consistent with the findings from the IMvigor210 cohort, two subclasses were identified within Cluster 2 in the GSE176307 cohort: Cluster 2-Sub1, showing immune activation accompanied by immunosuppressive signals, and Cluster 2-Sub2 characterized by low expression of both immune activation and suppressive signals (**Figures 7E-G**). These findings suggest that our SHAP-based clustering approach can reproducibly classify patient subclasses with distinct response profiles and prognostic outcomes in an independent cohort, highlighting its potential for clinical trials that delineate the heterogeneity of immunotherapy responses in diverse solid-tumor cancer types.

## 4 Discussion

In the present study, we developed a novel analysis framework combining SHAP-based interpretable feature extraction and spectral clustering to reveal intra-group heterogeneity in ICI responses among patients with mUC. We applied this approach to a discovery cohort (IMvigor210) and identified four distinct patient subclasses, each exhibiting unique combinations of genomic alterations, immune-related gene expression profiles, and clinical outcomes. Validation in an independent cohort (GSE176307) also showed similar clustering patterns. Although further investigation is necessary due to the cohort’s limited sample size and variable patient characteristics, these results suggest the potential of our framework to capture intra-group response heterogeneity, providing a basis for the development of subclass-specific therapeutic strategies in mUC.

Each subclass identified by our framework demonstrated distinct immunogenomic features that correlated with clinical outcomes. In the present study, Cluster 3, which showed the highest response rate (92.3%), was characterized by high PD-L1 expression, elevated expression of T-cell activation-related genes, frequent TP53 mutations, and favorable survival outcomes, representing a classic inflamed phenotype responsive to ICI (16, 24). In contrast, Cluster 1 (100% non-responders) presented with immune-desert phenotype, low TMB, frequent FGFR3 mutations, and elevated TGF-β signaling, all of which have been associated with poor ICI response in previous studies (25, 26).

Notably, Cluster 2 was characterized by high intra-group heterogeneity, as it contained two subgroups (Sub1 and Sub2) with contrasting immune characteristics and clinical outcomes, a pattern that was consistently observed in both IMvigor210 and GSE176307 cohorts. While Clusters 1 and 3 exhibited typical immunogenomic features associated with ICI response, Cluster 2 showed distinct behavior, leading to further consideration of the underlying biological mechanisms. Since the tumor microenvironment reflects these immune and genomic interactions, we next considered how the identified subgroups (Sub 1 and Sub 2) within Cluster 2 might be linked to potential tumor microenvironment mechanisms. Regarding immune dysfunction under conditions where immune activation coexists with immunosuppressive signaling, as shown in Sub 1, previous studies have suggested that T cell-related TGF-β signaling may be involved. Mariathasan et al. demonstrated that TGF-β signaling contributes to the exclusion of T cells from the tumor and leads to resistance to ICI response (16) while, similarly, Tauriello et al. reported that TGF-β promotes immune evasion and metastasis in colorectal cancer by modulating an immune-suppressive tumor microenvironment (27). These findings suggest that even high T-cell activation may not contribute to OS if coexisting immunosuppressive mechanisms (e.g., RB1 mutation, elevated TGF-β signaling, and low TMB) override effector immune responses and lead to progressive treatment resistance. In contrast, Sub 2 was characterized by the GU subtype (i.e., high TMB and low expression of immunosuppressive genes), despite harboring features generally associated with poor ICI response, such as an immune-desert phenotype, FGFR3 mutations, and low PD-L1 expression. In terms of the GU subtype, it is often associated with high TMB and DNA repair deficiencies, which may enhance immunogenicity and ICI responsiveness (28). Our collective findings therefore suggest that combining genomic and immune features through SHAP clustering better predicts ICI response, even in tumors with immune-desert phenotypes.

Based on these immunogenomic findings, for example, Cluster 1 (immune-desert with low PD-L1 expression, low TMB, and frequent FGFR3 mutations) could be more suitable for cytotoxic or targeted therapies, including antibody-drug conjugate (ADC) or erdafitinib (FGFR inhibitor) while Cluster 2-Sub 1 (immune activation and high immune suppression) may require combination approaches to modulate the tumor microenvironment, such as ICIs combined with TGF-β inhibitors. In contrast, Cluster 2- Sub 2, with high TMB, low immune suppression, and frequent FGFR3 mutations, could potentially benefit from combination therapy with ICIs and erdafitinib. Finally, Cluster 3, featuring strong immune activation, appears to represent the most immunotherapy responsive group, where ICIs could serve as the main therapeutic component. However, recent paradigm shifts in mUC treatment, such as the introduction of combination therapy with enfortumab vedotin plus pembrolizumab, have become the preferred first-line option (3, 29). Therefore, our findings, particularly specific genomic profiles identified in each cluster, should be interpreted in the context of these evolving treatment standards.

Our findings also highlight the clinical utility of machine learning techniques (e.g., SHAP) into transcriptomic analysis pipelines for mUC. Unlike conventional clustering methods, such as hierarchical clustering based on raw gene expression, SHAP values weight feature importance on outcome, enabling more informed subclassification. This method has been demonstrated in other cancers to better predict specific treatment response outcomes (30) and, together with prior studies (12–14), our results suggest that SHAP-based analyses can uncover patient-specific relationships between molecular profiles and clinical outcomes in mUC.

This study has several limitations. First, although we validated our findings in an external dataset (GSE176307), the cohort size was small, and patient characteristics differed from those in the IMvigor210 dataset. Therefore, further studies using larger and more diverse cohorts are needed to determine whether our clustering approach can be widely applied. Second, the use of atezolizumab as first-line treatment in the IMvigor210 cohort does not represent the current standard of care for mUC. Third, since our study is retrospective, a future prospective study is necessary to evaluate its clinical utility for patient stratification. Finally, although this study revealed biologically distinct subclasses, it remains unclear whether these findings directly reflect underlying immune mechanisms. Experimental validation through *in vitro* or *in vivo* models will be essential to confirm the biological relevance of the identified immunogenomic features.

In conclusion, our framework integrating SHAP-based interpretable feature extraction with spectral clustering uncovered subclass-level heterogeneity in ICI response, revealing biologically distinct immune subtypes in mUC that may guide the development of subclass-specific therapeutic strategies.

## Data Availability

The original contributions presented in the study are included in the article/**Supplementary Material**. Further inquiries can be directed to the corresponding author.

## Supplementary material

Supplementary Figure 1 Conventional clustering methods using 182 DEGs between responders and non-responders in the IMVigor210 cohort. (A) A k-means clustering heatmap showing the division of samples into two clusters. (E) A bar plot showing proportional distribution of responders and non-responders across the two clusters.
