# Supplementary figures and images for: Revealing intra-group immunotherapy response heterogeneity in metastatic urothelial carcinoma through interpretable feature extraction and spectral clustering
Source: Front Immunol. 2026 Jan 6;16:1629001. doi: 10.3389/fimmu.2025.1629001 (PMC12816215; doi:10.3389/fimmu.2025.1629001)

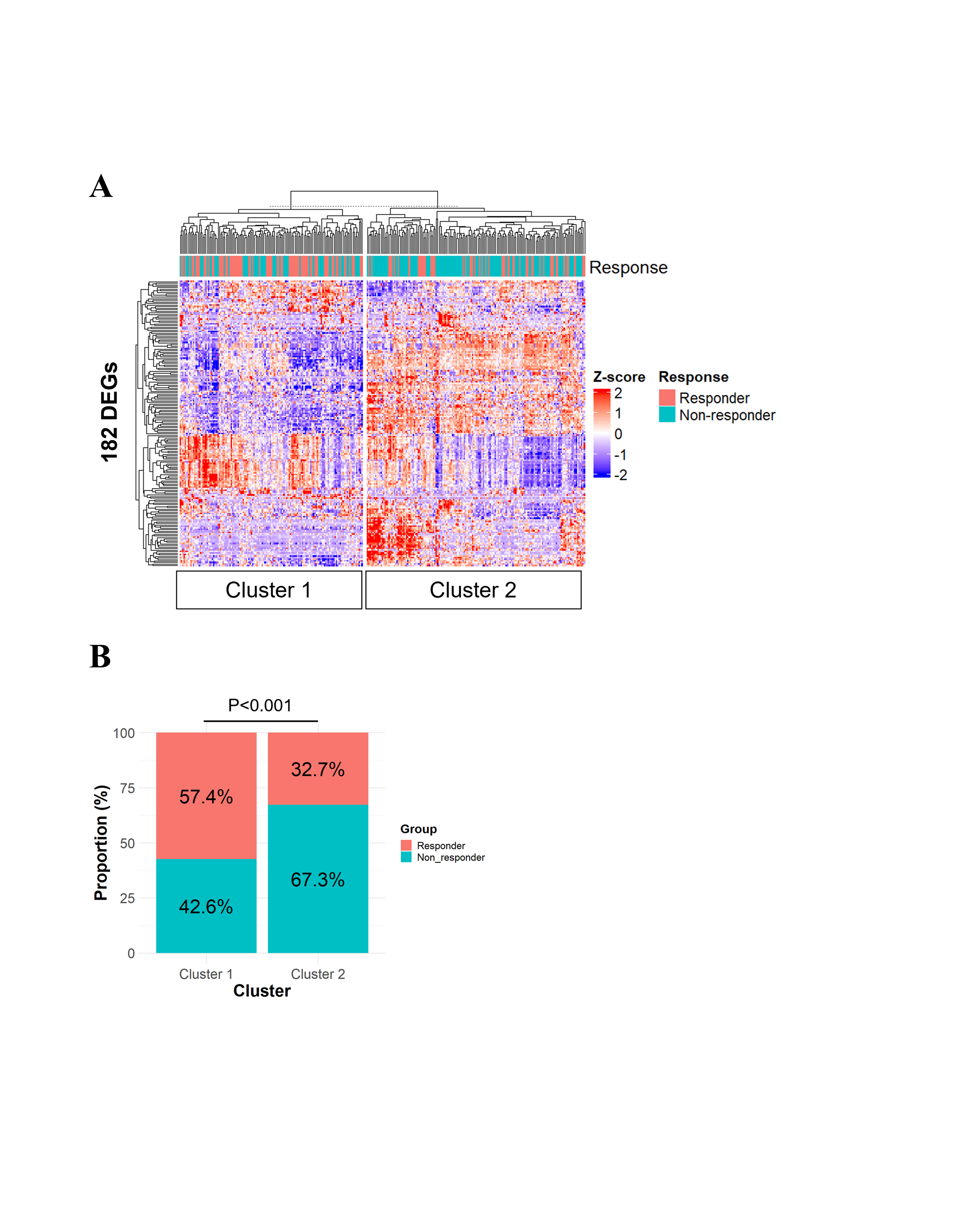

Supplement: Supplementary Figure 1 — Conventional clustering methods using 182 DEGs between responders and non-responders in the IMVigor210 cohort. (A) A k-means clustering heatmap showing the division of samples into two clusters. (E) A bar plot showing proportional distribution of responders and non-responders across the two clusters. [file Image1.tif]
